# Enhancing SVM for survival data using local invariances and weighting

**DOI:** 10.1186/s12859-020-3481-2

**Published:** 2020-05-19

**Authors:** Hector Sanz, Ferran Reverter, Clarissa Valim

**Affiliations:** 1 Department of Genetics, Microbiology and Statistics, Faculty of Biology, Universitat de Barcelona, Diagonal, 643, 08028 Barcelona, Catalonia Spain; 2 Centre for Genomic Regulation (CRG), The Barcelona Institute for Science and Technology, Dr. Aiguader 88, 08003 Barcelona, Spain; 3 Department of Global Health, Boston University, 801 Massachusetts Avenue, Boston, MA 02118 USA; 4 Department of Immunology and Infectious Diseases, Harvard T.H. Chen School of Public Health, 675 Huntington Ave, Boston, MA 02115 USA

**Keywords:** Support vector machines, Survival analysis, Kernel, Classification

## Abstract

**Background:**

The necessity to analyze medium-throughput data in epidemiological studies with small sample size, particularly when studying biomedical data may hinder the use of classical statistical methods. Support vector machines (SVM) models can be successfully applied in this setting because they are a powerful tool to analyze data with large number of predictors and limited sample size, especially when handling binary outcomes. However, biomedical research often involves analysis of time-to-event outcomes and has to account for censoring. Methods to handle censored data in the SVM framework can be divided into two classes: those based on support vector regression (SVR) and those based on binary classification. Methods based on SVR seem to be suboptimal to handle sparse data and yield results comparable to Cox proportional hazards model and kernel Cox regression. The limited work dedicated to assess methods based on of SVM for binary classification has been based on SVM learning using privileged information and SVM with uncertain classes.

**Results:**

This paper proposes alternative methods and extensions within the binary classification framework, specifically, a conditional survival approach for weighting censored observations and a semi-supervised SVM with local invariances. Using simulation studies and some real datasets, we evaluate those two methods and compare them with a weighted SVM model, SVM extensions found in the literature, kernel Cox regression and Cox model.

**Conclusions:**

Our proposed methods perform generally better under a wide variety of realistic scenarios about the structure of biomedical data. Specifically, the local invariances method using the conditional survival approach is the most robust method under different scenarios and is a good approach to consider as an alternative to other time-to-event methods. When analysing real data is a method to be considered and recommended since outperforms other methods in proportional and non-proportional scenarios and sparse data, which is something usual in biomedical data and biomarkers analysis.

## Background

Biomedical studies are oftentimes based on small sample sizes and on a medium to large number of variables. Support vector machine (SVM) models are a powerful tool to analyse this type of data because of their performance in analysis of sparse data, i.e., data with as many or more predictors than observations. SVMs have been widely applied for analysis of binary outcomes. As originally developed [1] these models are based on discriminating two classes of observations by a linear decision surface (hyperplane) and maximizing the distance between the hyperplane and the individual observations. If the classes are not separable by a linear surface, a non-linear transformation can be obtained through mapping the data on a different dimension space (feature space). This non-linear transformation can be obtained without explicitly mapping into the feature space through the use of a kernel function.

A common outcome in biomedical research is time-to-event. The challenge of analyzing time-to-event data is associated with occurrence of censoring as it is called the partially observed time-to-event of a participant whose follow-up ends before the event has occurred. There are different types of censoring but the most common is right censoring that occurs when an observation leaves the study before the end of follow-up or presenting an event, or when the study ends before the event has occurred. The most common traditional approach to analyze time-to-event data and handle censoring is the Cox proportional hazard regression [2]. This is a semi-parametric model based on a partial likelihood function (similar to the ordinary likelihood functions) that is defined in terms of the hazard function and assumes that: i) the baseline hazard is common to all observations; ii) linearity and additivity of the predictors with respect to log-hazard or log-cumulative hazard, and iii) proportionality of the hazards across predictor classes or constant hazard ratios over time. Another important requirement to obtain unbiased estimations with proportional hazards models is that the minimum number of events is at least 5 [3–5].

When the data is sparse, proportional hazards regression may not converge and yield unreliable and biased point estimates and statistical tests. Under sparsity, SVM or a kernelized (i.e., penalized) version of the Cox model [6] may be more appropriate. Generally, extensions of SVM to handle time-to-event and censored data can be based on a regression (SVR) or a classification approach (SVM). Most work has focused on SVR [7–9] and on a ranking (ordinal) methodology [10–12] and suggested that both approaches were comparable to proportional hazards model, in non-sparse scenarios, and the kernel Cox regression and, thus, may not provide any gains in accuracy of predictions. Only two methods have extended the SVM to survival data and handled censoring based on a binary classification approach: SVM learning using privileged information [13] (LUPI) and uncertain classes [14], proposed in Shiao and Cherkassky [15] work. In both methods, the censored data is basically weighted using the follow-up time without considering the overall probability of the event at the end of the follow-up period.

In this paper, we propose an alternative extension to allow SVM to model time-to-event data based on a binary classification SVM. To do that, we assign a probability to the censored data using a conditional survival approach considering the survival probability at each censored time. Moreover, we propose using a semi-supervised version of SVM with local invariances to model time-to-event data and compare the performance of the proposed approaches with the Cox proportional hazards regression, kernel Cox regression, and other SVM methods for survival analysis, such as LUPI and weighted SVM.

## Related work

### Traditional models for survival data – Kaplan Meier estimator and Cox proportional hazards model

In survival analysis, the non-negative time-to-event (be death or any other event) of a subject can be defined by the continuous random variable *T*^∗^. An important function related to the time-to-event data is the survival function *S*(*T*^∗^) = *P*(*T*^∗^ ≥ *t*^∗^), that is the probability of an individual to survive beyond time *t*^∗^. Due to censoring, *T*^∗^ is not observable but instead the pair (*T*, *δ*), where *T* is the time to censoring or to the event of interest and *δ* is the censoring indicator (0 for censored data and 1 for event).

The empirical survival function is an estimate of the survival function and is commonly obtained by the non-parametric Kaplan-Meier estimator [16]. This can be obtained applying the product:

1 $$ {\hat{S}}_{KM}(t)={\prod}_{i:{T}_{(i)}<t}\left(1-\frac{\delta_{(i)}}{n-i+1}\right) $$

, where *n* is the total number of individuals, *T*_(*i*)_ are the order statistics of the observed times for *i*-th observation and *δ*_(*i*)_ is the censoring indicator of *i*-th observation. The estimator in (1) is a decreasing step function that changes only at event times.

A second important function in analyses of time-to-event data is the hazard function, being the Cox proportional hazards model [2] the most popular model used in analysis of survival data. It is defined in terms of the hazard function:

2 $$ \lambda \left(t|{\boldsymbol{x}}_i\right)={\lambda}_0(t)\exp \left(\left\langle {\boldsymbol{x}}_i,\boldsymbol{\beta} \right\rangle \right) $$

where *λ*(*t*| ***x***_*i*_) is the hazard at time *t* of an observation *i* with covariates vector x_i_, λ_0_(t) is the baseline hazard function, β is the vector of coefficients of the model and 〈x_i_, β〉 is the dot product between x_i_ and β, i.e., the linear predictor function. The model assumes a baseline hazard that is common to all observations in the study population. In this model, the hazard of a subject increases multiplicatively with covariates.

In the Cox proportional hazards model, the baseline hazard is modelled semi-parametrically, i.e., the baseline hazard does not need to be specified and the optimization function is based on a partial likelihood. The Cox model is more robust to outliers than other models because it uses only the rank ordering of the failure and censoring times. The partial likelihood accounting for censored observations can be expressed as:

3 $$ L\left(\boldsymbol{\beta} \right)={\prod}_{i=1}^n{\left(\frac{\exp \left(\left\langle {\boldsymbol{x}}_i,\boldsymbol{\beta} \right\rangle \right)}{\sum_{j\in {R}_i}\exp \left(\left\langle {\boldsymbol{x}}_i,\boldsymbol{\beta} \right\rangle \right)}\right)}^{\delta_i} $$

where *R*_*i*_ is the set of individuals at risk of having an event at time *t*_*i*_, *δ*_*i*_ the censoring indicator of the observation with time *t*_*i*_ and ***x***_***i***_ vector of covariates of observation *i*. Applying the logarithm transformation to the partial likelihood we obtain the log partial likelihood, which is maximized through Newton-Raphson algorithm. The maximum partial likelihood estimator is asymptotically unbiased, efficient and normally distributed [17].

### Kernel Cox regression

This is a penalized version of the Cox model, in which a kernel is added to model the hazard as a function of covariates. For the general Cox model for observation *i*, at time *t*, with a vector of covariates ***x***_*i*_, the hazard can be expressed as

4 $$ \lambda \left(t|{\boldsymbol{x}}_i\right)={\lambda}_0(t)\ \exp \left(f\left({\boldsymbol{x}}_{\boldsymbol{i}}\right)\right) $$

where *λ*_0_(*t*) is the unspecified baseline hazard function and *f*(***x***_***i***_) is an arbitrary function. Li and Luan [6] proposed using the log partial likelihood as a loss function and reformulate the problem as finding the function *f* in the penalized log-likelihood such that

5 $$ \log \left(L(f)\right)={\sum}_{i=1}^n{\delta}_i\ \left(f\left({\boldsymbol{x}}_{\boldsymbol{i}}\right)-\mathit{\log}\ {\sum}_{j\in {R}_i}\exp \left(f\left({\boldsymbol{x}}_j\right)\right)\right)+\xi\ {\left\Vert\ f\right\Vert}_H^2 $$

where *f* is assumed to be from a Reproducing Kernel Hilbert Space, *H*, defined by a kernel function and a *ξ* > 0 regularization parameter [18]. The solution to this problem is given by the representer theorem [18] where the optimal *f*(***x***) has the form

6 $$ f\left(\boldsymbol{x}\right)={\sum}_{i=1}^n{\alpha}_ik\left(\boldsymbol{x},{\boldsymbol{x}}_{\boldsymbol{i}}\right)+b $$

The optimal ***α*** = (*α*_1_, …, *α*_*n*_) in (6) can be found by plugging (6) into (5), resulting in a convex optimization problem to which the solution can be found by any unconstrained optimization method. The term *b* is the intercept or bias usually computed as the average error between the target and predicted value.

### Survival analysis using the SVM based on binary classification

Two approaches have been proposed in this class of models by Shiao and Cherkassky [15]: the LUPI approach developed by Vapnik and Vashist [13] and the SVM with uncertain classes developed by Niaf et al. [14]. LUPI uses the censoring information as privileged information (only available for the training data) and, thus, includes additional information in the training process to enrich the learning process. Two different spaces are described, the decision space and the correcting space (the one with the censoring information). SVM with uncertain classes allows to define less than perfectly the belonging class of observations, i.e., it allows some degree of confidence regarding the class.

Shiao and Cherkassky suggested measuring privileged information for LUPI and the SVM uncertainty using the proportion of follow-up time with which an *i*-th censored subject contributes. Therefore, for the censored observation *i*, the weight or probability assigned to the observation is ${W}_i=\frac{{\mathrm{T}}_{\mathrm{i}}}{\uptau}$, being *τ* the maximum follow-up time in the study cohort. For the event this value is fixed to be 0.

#### LUPI SVM

The LUPI approach is based on a triplet $\left({\boldsymbol{x}}_i,{\boldsymbol{x}}_i^{\ast },{y}_i\right)$ for *i* = 1, …, *n* observations, where ${\boldsymbol{x}}_i\in {\mathbb{R}}^d,{\boldsymbol{x}}_i^{\ast}\in {\mathbb{R}}^k$ and *y*_*i*_ ∈ {±1}. The (***x***_*i*_, *y*_*i*_) are the usual training data and ${\boldsymbol{x}}_i^{\ast }$ defines the privileged information only present in the training data, i.e., the information (variables) only present when modelling the data. The privileged information is not available when predicting the class of a new observation. In the LUPI approach two different spaces are described: i) the space related to ***x***, known as decision space, which is the same feature space used in standard SVM and ii) the space related to ***x***^∗^, known as correcting space, which contains the privileged information about the training data and not available for predictions of future observations. The LUPI estimates the decision function and corrects it using the correcting function via privileged information. The main optimization problem is expressed as in equation (7).

7 $$ {\displaystyle \begin{array}{l}\underset{\boldsymbol{w},{\boldsymbol{w}}^{\ast },b,{b}^{\ast }}{\operatorname{minimize}}\kern1em \frac{1}{2}\left({\left\Vert \boldsymbol{w}\right\Vert}^2+\gamma {\left\Vert {\boldsymbol{w}}^{\ast}\right\Vert}^2\right)+C\sum \limits_{i=1}^n{\xi}_i\\ {}\mathrm{subject}\kern0.5em \mathrm{to}\kern0.5em {\xi}_i=\left(\left\langle {\boldsymbol{w}}^{\ast },{\boldsymbol{x}}_i^{\ast}\right\rangle +{b}^{\ast}\right),\kern14em i=1,\dots, n\\ {}{y}_i\left(\left\langle \boldsymbol{w},{\boldsymbol{x}}_i\right\rangle +b\right)\ge 1-\left(\left\langle {\boldsymbol{w}}^{\ast },{\boldsymbol{x}}_i^{\ast}\right\rangle +{b}^{\ast}\right),\kern8.2em i=1,\dots, n\\ {}\left(\left\langle {\boldsymbol{w}}^{\ast },{\boldsymbol{x}}_i^{\ast}\right\rangle +{b}^{\ast}\right)\ge 0,\kern13.8em i=1,\dots, n\end{array}} $$

where ***w*** is the weight vector of the separating hyperplane**,** ***x***_*i*_ is the vector of covariates for subject *i*, *ξ*_*i*_ are the slack variables and *b* is the bias term of the hyperplane of the decision space. The analogous parameters, ***w***^***∗***^**,** ${\boldsymbol{x}}_i^{\ast }$ and *b*^∗^ are in the correcting space.

The decision function and the correcting functions depend on the decision and correcting space respectively. Although, the decision function has the same expression of the usual SVM, the coefficients of the LUPI decision function depend on kernels in both spaces. The SVM and the LUPI solutions are exactly the same when the privileged information is rejected (when γ tends to 0 in expression (7).

The time to follow-up and time-to-event are observable and known in the training set but not in the test set. Thus, the censoring information that is only present in the training set can be used as privileged information. Shiao and Cherkassky proposed using the pair (*T*_*i*_, *W*_*i*_) as the privileged information.

#### Uncertainty SVM

This method allows defining less than perfectly some observations, assigning them an uncertainty in their class. For these uncertainties a confidence level or probability regarding the class is provided. We will refer to the Uncertainty SVM onward in this manuscript as pSVM (probabilistic SVM).

The pSVM assigns observations to a class through a hinge loss and estimates probability of belonging to the class through the *ϵ*-insensitive cost function. Given an observation *i*, we define the pair (***x***_*i*_, *l*_*i*_) as the training set of input vectors along with their corresponding group of classes. These classes can be defined as

8 $$ {\displaystyle \begin{array}{l}{l}_{\mathrm{i}}={y}_i\in \left\{\pm 1\right\}\kern2em \mathrm{for}\ i=1,\dots, n\\ {}{l}_i={p}_i\in \left[0,1\right]\kern2.1em \mathrm{for}\ i=n+1,\dots, m\end{array}} $$

where *n* is the number of observations with known classes (perfectly definite), (*m* − *n* − 1) is the number of observations with uncertain classes, and *p*_*i*_ is the uncertainty associated with ***x***_*i*_ in a regression setting. More specifically, the posterior probability for class 1 is given by

9 $$ {p}_i= Prob\left({Y}_i=1|{\boldsymbol{X}}_i={\boldsymbol{x}}_i\right) $$

The resulting associated optimization problem is

10 $$ {\displaystyle \begin{array}{l}\underset{\boldsymbol{w},b}{\operatorname{minimize}}\kern1em \frac{1}{2}{\left\Vert \boldsymbol{w}\right\Vert}^2\\ {}\mathrm{subject}\kern0.5em \mathrm{to}\kern2em {y}_i\left(\left\langle \boldsymbol{w},{\boldsymbol{x}}_i\right\rangle +b\right)\ge 1,\kern4em i=1,\dots, n\\ {}\kern6em {z}_i^{-}\le \left\langle \boldsymbol{w},{\boldsymbol{x}}_i\right\rangle +b\le {z}_i^{+},\kern3em i=n+1,\dots, m\end{array}} $$

where ***w*** is the weight vector of the hyperplane**,** ***x***_*i*_ is the vector of covariates for subject *i*, *b* is the bias term of the hyperplane, and *y*_*i*_ is the class of subject *i*. The terms ${z}_i^{-}$ and ${z}_i^{+}$ are boundaries depending on *p*_*i*_. If *n* = *m* the problem is reduced to a hard margin SVM. To allow misclassification in classes, slack variables ${\xi}_i,{\xi}_i^{-}\ \mathrm{and}\ {\xi}_i^{+}$ are introduced and the optimization problem expressed in (10) can be rewritten as

11 $$ {\displaystyle \begin{array}{l}\underset{\boldsymbol{w},\boldsymbol{\xi}, {\boldsymbol{\xi}}^{-},{\boldsymbol{\xi}}^{+},b}{\operatorname{minimize}}\kern1em \frac{1}{2}{\left\Vert \boldsymbol{w}\right\Vert}^2+C\sum \limits_{i=1}^n{\xi}_i+\tilde{C}\sum \limits_{i=n+1}^m\left({\xi}_i^{-}+{\xi}_i^{+}\right)\\ {}\mathrm{subject}\kern0.5em \mathrm{to}\kern2em {y}_i\left(\left\langle \boldsymbol{w},{\boldsymbol{x}}_i\right\rangle +b\right)\ge 1-{\xi}_i,\kern5em i=1,\dots, n\\ {}\kern6em {z}_i^{-}-{\xi}_i^{-}\le \left\langle \boldsymbol{w},{\boldsymbol{x}}_i\right\rangle +b\le {z}_i^{+}+{\xi}_i^{+},\kern2em i=n+1,\dots, m\\ {}\kern6em {\xi}_i\ge 0,\kern11.5em i=1,\dots, n\\ {}\kern6em {\xi}_i^{-}\ge 0,\kern11em i=n+1,\dots, m\\ {}\kern6em {\xi}_i^{+}\ge 0,\kern11em i=n+1,\dots, m\end{array}} $$

The proportional follow-up time approach computes the probability *p*_*i*_ for censored data, and subsequently ${z}_i^{-}$ and ${z}_i^{+}$, as $\frac{{\mathrm{T}}_{\mathrm{i}}}{\uptau}$, being *τ* the maximum follow-up time established in the study cohort. For an event, this value is fixed to be 0.

#### Weighted SVM

Another approach that has not been tested in the literature is to address the survival-SVM as a weighted SVM (wSVM) problem (see eq. 12). The basic idea of wSVM is to assign to each observation a different weight according to its relative importance in the class such that different data points contribute differently to the learning of the decision surface [19]. This methodology is particularly useful to handle outliers because upon detecting an outlier, we can diminish its effect in the estimation of the separating hyperplane.

12 $$ {\displaystyle \begin{array}{l}\underset{\boldsymbol{w},\boldsymbol{\xi}}{\operatorname{minimize}}\kern1.5em \frac{1}{2}{\left\Vert \boldsymbol{w}\right\Vert}^2+C\sum \limits_{i=1}^n{W}_i{\xi}_i\\ {}\mathrm{subject}\kern0.5em \mathrm{to}\kern1em {y}_i\left(\left\langle \boldsymbol{w},{\boldsymbol{x}}_i\right\rangle +b\right)\ge 1-{\xi}_i,\kern10.5em i=1,\dots, n\\ {}{\xi}_i\ge 0,\kern17em i=1,\dots, n\end{array}} $$

where *W*_*i*_ is the weight or probability of each observation. The censored observation can be seen as a partial or weighted observation because, an observation censored just at the beginning of the study, for instance, is adding no information to the data and have weight close to 0. A censored observation just before the end of the follow-up period should be treated almost as complete observation (a weight close to 1).

## Proposed approaches

### Proposed weighting methods

Censored data has been handled through assigning a weight or probability to an observation assuming proportionality of follow-up time, i.e., linearly associated with the observed follow-up period. The approach consists in computing the weight as ${\mathrm{W}}_{\mathrm{i}}=\frac{{\mathrm{T}}_{\mathrm{i}}}{\uptau}$, being τ the maximum follow-up time in the study cohort and T_i_ the censored time for an observation *i*. For the events, this value is fixed and is equal to non-events who completed the follow-up period,i.e., subjects who were free of events by the end of the study period. Therefore, this method does not account for the overall survival probability. Use of this information in time-to-event data is important because, for example, a censored observation at the beginning of the follow-up will be more likely to have an event if the overall survival probability is 0.1 than if it is 0.9. The current proposed methods in the SVM literature do not use this information because of the proportionality on follow-up time approach. We propose weighting the censored observations based on the probability of surviving *t*_*i*_ + *z* years (13) (conditional survival probability), given that the participant *i* is still alive at *t*_*i*_ (censored time) that can be estimated through the Kaplan-Meier estimator as in eq. (1).

13 $$ {\hat{S}}_z\left({t}_i\right)=\frac{\hat{S}\left({t}_i+z\right)}{\hat{S}\left({t}_i\right)} $$

This modification would improve the accuracy of the method by including in the weighting process information about the overall survival probability of the cohort and the survival curve shape. More specifically, our proposal is to weight (or to assign a probability to be an event or non-event) the censoring information using the conditional survival probability in the following way for each specific SVM method:

- For the LUPI method our proposal is to define the weight (importance) of the privileged information based on the Kaplan-Meier estimation of eq. (12), i.e., ${\boldsymbol{x}}_i^{\ast }={\hat{S}}_z\left({t}_i\right)=\frac{\hat{S}\left({t}_i+z\right)}{\hat{S}\left({t}_i\right)}$
- For pSVM our proposal is to compute the uncertain probability of the censored data based on the conditional probability of having the event using the Kaplan-Meier estimator (13).
- For WSVM our proposal, following the same idea than the previous methods, is using a conditional survival approach, based on the Kaplan-Meier estimator of (13). Events and non-events at the end of follow-up time will have a weight of 1.

### Local invariances SVM

Alternatively, censoring can be treated as a semi-supervised problem, an approach that has not been considered in the SVM literature. In the semi-supervised setting, there are observations with class and others with class unknown. The goal is to learn from both types of data to find the decision surface that separates both classes. We propose to treat censored observations as unknown classes, i.e., observations we don’t know their event status within the follow-up period, and events and non-events at the end of follow-up as known classes, i.e., observations with known event status.

In the non-SVM specific literature [20], a framework has been proposed of semi-supervised learning in the reproducing kernel Hilbert space *H* (RKHS) associated with a given kernel function *k*, using local invariances that explicitly characterize the behaviour of the target function around both known and unknown data. Three types of invariances have been proposed: i) invariance to small changes in the observations, restricting the gradient of the function to be small at the observed data; ii) invariance to averaging across a small neighbourhood around observations, restricting the function value at each observation to be similar to the average value around a small neighbourhood of the corresponding observation; and iii) invariances to local transformation, like rotational and translational invariance (specially focused in problems such as handwritten digit recognition and vision problems). The third invariance is not relevant for survival analysis. The optimization problem (14) includes the hinge-loss for known data and the *ϵ*-insensitive loss for unknown data to obtain a semi-supervised SVM with local invariances (inSVM).

14 $$ {\displaystyle \begin{array}{l}\underset{g,b}{\operatorname{minimize}}\kern1.5em \frac{1}{2}{\left\Vert g\right\Vert}^2+\sum \limits_{i=l+1}^n\left({\xi}_i+{\xi}_i^{\ast}\right)+\sum \limits_{\mathrm{i}=1}^1{\gamma}_i\\ {}\mathrm{subject}\kern0.5em \mathrm{to}\kern1em -\left\langle g,{z}_i\right\rangle -b\le \in -{\xi}_i,\kern14.5em i=l+1,\dots, n\\ {}\left\langle g,{z}_i\right\rangle +b\le \in +{\xi}_i^{\ast },\kern14.2em i=l+1,\dots, m\\ {}{\xi}_i\ge 0,\kern19.7em i=l+1,\dots, n\\ {}{\xi}_i^{\ast}\ge 0,\kern19.2em i=l+1,\dots, n\\ {}{y}_i\left(\left\langle g,\phi \left({\boldsymbol{x}}_i\right)\right\rangle +b\right)\ge 1-{\gamma}_i,\kern11.4em i=1,\dots, l\\ {}{\gamma}_i\ge 0,\kern19.5em i=1,\dots, l\end{array}} $$

where *g* ∈ *H* is the target function and *z*_*i*_ is the representer of the functional associated with the invariance [20] . In particular, given the following expression of the Gaussian kernel

15 $$ k\left({\boldsymbol{x}}_1,{\boldsymbol{x}}_2\right)=\exp \left(-\frac{1}{2{\sigma}^2}{\left\Vert {\boldsymbol{x}}_1-{\mathbf{x}}_2\right\Vert}^2\right) $$

where *σ* is the parameter of the Gaussian kernel, the evaluation functional of the representer of the derivative functional ${L}_{x_{i,j}}(f)={\left.\frac{\partial f}{\partial {x}^j}\right|}_{{\boldsymbol{x}}_i}$, for any *f* in the RKHS *H* associated with the Gaussian kernel is:

16 $$ {z}_{x_{i,j}}\left(\boldsymbol{x}\right)=\frac{1}{\sigma^2}\left({x}^j-{x}_i^j\right)\mathit{\exp}\left(-\frac{1}{2{\sigma}^2}{\left\Vert \boldsymbol{x}-{\boldsymbol{x}}_i\right\Vert}^2\right) $$

and the dot product between two representers of the functional derivative is expressed as:

17 $$ \left\langle {Z}_{x_{i,j}},{Z}_{x_{p,q}}\right\rangle =\left\{\begin{array}{ll}-\frac{1}{\sigma^4}\left({x}_i^j-{x}_p^j\right)\left({x}_i^q-{x}_p^q\right)\exp \left(-\frac{1}{2{\sigma}^2}{\left\Vert {\boldsymbol{x}}_i-{\boldsymbol{x}}_p\right\Vert}^2\right)& \mathrm{if}\kern0.5em j\ne q\\ {}\frac{1}{\sigma^4}\left({\sigma}^2-{\left({x}_i^j-{x}_p^j\right)}^2\right)\exp \left(-\frac{1}{2{\sigma}^2}{\left\Vert {\boldsymbol{x}}_i-{\boldsymbol{x}}_p\right\Vert}^2\right)& \mathrm{if}\kern0.5em j=q\end{array}\right. $$

where *i* and *p* are the subject indices and *j* and *q* are the indices of the specific variable in the specific ***x*** vector.

Another type of local invariance is the local averaging. So, considering the Gaussian kernel in (15) and the following Gaussian density

18 $$ p\left(\boldsymbol{x}\right)=\frac{1}{{\left(2\pi \right)}^{\frac{d}{2}}\ {\sigma}_p^d}\exp \left(-\frac{1}{2{\sigma}_p^2}{\left\Vert \boldsymbol{x}\right\Vert}^2\right) $$

and given that the convolution of two Gaussian densities is a Gaussian density, the representer of the local averaging functional, ${L}_{x_i}(f)={\int}_Xf(u)p\left({x}_i-u\right) du-f\left({x}_i\right)$, for any *f* in the RKHS *H* associated with the Gaussian kernel, shall be expressed as:

19 $$ {z}_{{\boldsymbol{x}}_i}\left(\boldsymbol{x}\right)=\frac{\sigma_k^d}{{\left({\sigma}_k+{\sigma}_p\right)}^d}\mathit{\exp}\left(-\frac{1}{2{\left({\sigma}_k+{\sigma}_p\right)}^2}{\left\Vert {\boldsymbol{x}}_i-\boldsymbol{x}\right\Vert}^2\right)-\exp \left(-\frac{1}{2{\sigma}_k^2}{\left\Vert {\boldsymbol{x}}_i-\boldsymbol{x}\right\Vert}^2\right) $$

and the dot product between two representers of the averaging functional

20 $$ \left\langle {z}_{{\boldsymbol{x}}_i},{z}_{{\boldsymbol{x}}_j}\right\rangle =\frac{\sigma_k^d}{{\left({\sigma}_k+2{\sigma}_p\right)}^2}\mathit{\exp}\left(-\frac{1}{2{\left({\sigma}_k+2{\sigma}_p\right)}^2}{\left\Vert {\boldsymbol{x}}_i-{\boldsymbol{x}}_j\right\Vert}^2\right)-\frac{\sigma_k^d}{{\left({\sigma}_k+{\sigma}_p\right)}^d}\mathit{\exp}\left(-\frac{1}{2{\left({\sigma}_k+{\sigma}_p\right)}^2}{\left\Vert {\boldsymbol{x}}_i-{\boldsymbol{x}}_j\right\Vert}^2\right)-{z}_{{\boldsymbol{x}}_j}\left({\boldsymbol{x}}_i\right) $$

where *σ*_*k*_ and *σ*_*p*_ are the sigma values specified for the Gaussian kernel and Gaussian density respectively, *d* is the number of covariates and ${z}_{x_j}\left({\boldsymbol{x}}_i\right)$ is as defined in eq. (19).

Calculations and proofs associated with the inSVM methodology can be found in Lee et al. [20] .

### Implementation

The SVM methods presented in this paper: LUPI, pSVM, wSVM and inSVM, had not been implemented in the widely used R software [21]. Therefore, we have written R functions that will be included in a R package.

## Simulation studies

We conducted simulation studies to compare the proposed approaches in different scenarios. Simulations included varying sample size (50 and 300 subjects), 30 predictor variables (or features), and a proportional and non-proportional hazard of comparison groups. Moreover we varied the proportion of censoring (10–30%) and the distribution of the follow-up time (uniform, positive skewed and negative skewed). Those choices were based on realistic scenarios encountered in data we previously analysed. Based on the proportional hazards framework, the time-to-event was generated using the Gompertz distribution.

Specifically, the 30 predictor variables were generated following a multivariate normal distribution with mean defined by a realization of an uniform distribution U (0.03,0.06). The variables were classified in four groups according to their pairwise correlation: no correlation (around 0), low correlation (around 0.2), medium correlation (around 0.5) and high correlation (around 0.8). These four levels of correlation reflected correlation of predictors in the biomedical field such as transcriptional profile or the inflammatory process. These variables were used to compare two scenarios of time-to-event data using the Cox proportional hazards model. In the proportional hazards framework the time-to-event variable can be generated, based on the Gompertz distribution [22] as

21 $$ T=\frac{1}{\alpha}\left(1-\frac{\alpha \log (U)}{\gamma \exp \left(\left\langle \boldsymbol{\beta}, {\boldsymbol{x}}_i\right\rangle\ \right)}\right) $$

where *U* follows a Uniform (0,1) distribution, ***β*** is the vector of coefficients associated with each variable, and *α* ∈ (−∞, ∞) and *γ* > 0 are the scale and shape parameters, respectively, of the Gompertz distribution. The values for these parameters were selected so that overall survival was around 0.6 at 18 months follow-up time.

To generate scenarios in which the hazard of comparison groups was not proportional, a noise has been added into the exp(〈***β***, ***x***_*i*_〉) term in eq. (21), forcing the hazard to be a shared frailty model [23]. The frailty was chosen so that there were 5 groups of observations with same size that shared a common frailty.

### Tuning parameters and test performance

For the cost parameters *C* and $\overset{\sim }{C}$, we selected the values 0.1, 1, 10, and 100, and for the Gaussian kernel parameters *σ* the values 0.25, 0.5, 1, 2, and 4. A two-step approach was used to estimate tuning parameters and evaluate operational characteristics of the SVM models using the best combination of tuning parameters: in the first step, for each combination of parameters, 10 training datasets were fitted and each of them was validated using 10 different validation datasets. The combination of parameters with largest accuracy was used to measure the performance of the models in the second step. In the second step, new 10 datasets were simulated for estimation of models given the best combination of tuning parameters found in the first step and for each of those, 10 testing datasets were simulated to compare the performance of the SVM models based on the following metrics: accuracy (proportion of correctly classified observations), Matthews’ correlation, normalized mutual information, area under the ROC curve (AUC-ROC), sensitivity, specificity and F1 score. Therefore, 100 datasets have been tested and used to compute the mean and the standard deviation of the metrics used as a summary performance of each method.

## Real-life datasets

We applied our approaches to three datasets from the “Survival” package available in the R software repository [17]. Parameters were tuned and the accuracy, AUC-ROC, sensitivity, specificity and F1 score was estimated through a 5-fold nested-cross validation repeating the process in 10 resampled datasets. The follow-up time was censored to the third quartile of the maximum observed follow-up time in each dataset. We used the same analytical methods and the same grid of tuning parameter values of the simulation studies described above. Briefly, datasets of the following studies were analyzed:

- Lung Study: this study was conducted by the North Central Cancer Treatment Group (NCCTG) and aimed to estimate the survival of patients with advanced lung cancer. The available dataset was comprised of 167 observations, 89 events during the follow-up time of 420 days, and 10 variables. A total of 36 observations were censored before the end of follow-up. The overall survival probability at the end of follow-up period was 0.40.
- Stanford2 Study: this dataset was extracted from the Stanford Heart Transplant study and was comprised of 157 observations, 4 variables and the maximum follow-up time is 1264 days. A total of 88 events were recorded and 29 observations were censored before the end of follow-up. The overall survival probability at the end of follow-up period was 0.41.
- PBC Study: this study was nested in the Mayo Clinic trial of primary biliary liver cirrhosis (PBC) that was conducted between 1974 and 1984. A total of 424 PBC patients were referred to Mayo Clinic during the ten-year interval and met eligibility criteria for the randomized placebo controlled trial of the drug D-penicillamine. The data subset used in the current paper contains 258 observations and 22 variables. From the whole cohort 93 observations experienced the event, 65 finalized the follow-up period without presenting an event and 100 were censored before the end of the follow-up time of 2771 days. The overall survival probability at the end of follow-up period was 0.57.

## Results

### Simulated datasets

In the four simulated scenarios with a sample size of 300 in which hazards of comparison groups were proportional, the Cox proportional hazards model and pSVM (linear kernel) performed comparably to inSVM (gradient and averaging). Specifically, the accuracy was 0.89 for the Cox proportional hazards model, 0.87 for the linear pSVM and 0.84 for inSVM (Table 1). The AUC-ROC of the three models ranged from 0.92 to 0.96. Generally, the distribution and proportion of censoring did not affect results, with the inSVM-gradient being the most sensitive to the proportion of observations that were censored. LUPI methods (proposed Kaplan-Meier and proportional approach) performed similarly to pSVM using a radial kernel. The accuracy for a 10 and 30% censoring was 0.77.

**Table 1:** Accuracy results in a 300 observations proportional hazards, zero skew, 10 and 30% censoring. Prediction accuracy of all tested approaches when simulated data was generated with 300 observations and the following assumptions: proportional hazards, zero skew, 10 and 30% censoring. The table summarizes the mean (and standard deviation) of the following metrics: accuracy, Matthews’ correlation, normalized mutual information (NMI), area under the Receiver Operating Characteristic curve (AUC-ROC), sensitivity (Sn), specificity (Sp) and F1-score (F1)

	10% censoring							30% censoring						
Method	Accuracy	Matthews	NMI	AUC-ROC	Sn	Sp	F1	Accuracy	Matthews	NMI	AUC-ROC	Sn	Sp	F1
**Cox model**	0.89 (0.02)	0.78 (0.03)	0.50 (0.05)	0.96 (0.01)	0.61 (0.04)	0.60 (0.04)	0.60 (0.04)	0.89 (0.02)	0.79 (0.04)	0.51 (0.06)	0.96 (0.01)	0.61 (0.03)	0.60 (0.04)	0.60 (0.04)
**Kernel Cox**	0.81 (0.02)	0.62 (0.05)	0.30 (0.05)	0.88 (0.02)	0.42 (0.01)	0.91 (0.03)	0.50 (0.01)	0.80 (0.02)	0.59 (0.04)	0.26 (0.05)	0.86 (0.02)	0.32 (0.01)	0.93 (0.03)	0.52 (0.01)
**wSVM-KM**	0.75 (0.03)	0.50 (0.06)	0.19 (0.05)	0.87 (0.02)	0.40 (0.01)	0.95 (0.03)	0.54 (0.01)	0.68 (0.02)	0.39 (0.05)	0.11 (0.03)	0.86 (0.03)	0.39 (10.25)	0.95 (0.03)	0.55 (0.1)
**wSVM-Prop**	0.75 (0.03)	0.50 (0.06)	0.18 (0.05)	0.87 (0.02)	0.39 (0.01)	0.95 (0.03)	0.53 (0.01)	0.68 (0.02)	0.38 (0.05)	0.11 (0.03)	0.85 (0.02)	0.39 (0.01)	0.92 (0.03)	0.54 (0.1)
**pSVM-linear-KM**	0.88 (0.02)	0.73 (0.04)	0.46 (0.05)	0.95 (0.01)	0.85 (0.03)	0.84 (0.03)	0.81 (0.02)	0.88 (0.02)	0.72 (0.05)	0.43 (0.07)	0.95 (0.02)	0.87 (0.04)	0.87 (0.03)	0.82 (0.02)
**pSVM-linear-prop**	0.87 (0.02)	0.73 (0.04)	0.45 (0.05)	0.95 (0.01)	0.85 (0.04)	0.85 (0.03)	0.80 (0.03)	0.86 (0.02)	0.72 (0.05)	0.42 (0.07)	0.94 (0.02)	0.86 (0.04)	0.86 (0.03)	0.80 (0.03)
**pSVM-radial-KM**	0.79 (0.02)	0.57 (0.05)	0.25 (0.05)	0.88 (0.02)	0.69 (0.07)	0.88 (0.04)	0.74 (0.05)	0.79 (0.02)	0.58 (0.04)	0.27 (0.04)	0.86 (0.02)	0.67 (0.07)	0.88 (0.04)	0.74 (0.05)
**pSVM-radial-prop**	0.77 (0.02)	0.57 (0.05)	0.24 (0.05)	0.88 (0.02)	0.67 (0.07)	0.85 (0.04)	0.72 (0.05)	0.77 (0.02)	0.58 (0.04)	0.27 (0.04)	0.86 (0.02)	0.69 (0.03)	0.87 (0.05)	0.74 (0.05)
**LUPI-linear-KM**	0.78 (0.03)	0.56 (0.05)	0.28 (0.05)	0.84 (0.03)	0.81 (0.04)	0.74 (0.04)	0.75 (0.03)	0.77 (0.02)	0.55 (0.05)	0.27 (0.06)	0.84 (0.03)	0.81 (0.04)	0.74 (0.04)	0.77 (0.03)
**LUPI-linear-prop**	0.77 (0.03)	0.55 (0.05)	0.28 (0.05)	0.84 (0.03)	0.81 (0.04)	0.74 (0.04)	0.75 (0.03)	0.77 (0.02)	0.55 (0.05)	0.27 (0.06)	0.84 (0.03)	0.81 (0.04)	0.73 (0.04)	0.75 (0.03)
**inSVM-gradient**	0.84 (0.02)	0.68 (0.05)	0.37 (0.06)	0.92 (0.02)	0.87 (0.04)	0.90 (0.03)	0.84 (0.04)	0.80 (0.02)	0.60 (0.05)	0.28 (0.05)	0.89 (0.02)	0.87 (0.04)	0.90 (0.03)	0.84 (0.04)
**inSVM-averaging**	0.83 (0.02)	0.66 (0.05)	0.35 (0.06)	0.92 (0.02)	0.88 (0.04)	0.89 (0.03)	0.84 (0.04)	0.83 (0.02)	0.66 (0.05)	0.35 (0.06)	0.92 (0.02)	0.88 (0.04)	0.89 (0.03)	0.84 (0.04)

Conversely, when the sample size was decreased to 50, the proportion of censored observations affected all metrics of predictive accuracy even for data simulated meeting the proportional hazards assumption (Table 2). pSVM, inSVM and kernel Cox regression had the best performance in the 10% censoring scenario with an accuracy of approximately 0.75. The Cox model, wSVM and pSVM-radial had the worse performance with an accuracy of 0.62–0.67. Predictive accuracy was slightly decreased with increases in the proportion of censoring to 30% except for wSVM.

**Table 2:** Accuracy results in a 50 observations proportional hazards, zero skew, 10 and 30% censoring. Prediction accuracy of all tested approaches when simulated data was generated with 50 observations and the following assumptions: proportional hazards, zero skew, 10 and 30% censoring. The table summarizes the mean (and standard deviation) of the following metrics: accuracy, Matthews’ correlation, normalized mutual information (NMI), area under the Receiver Operating Characteristic curve (AUC-ROC), sensitivity (Sn), specificity (Sp) and F1-score (F1)

	10% censoring							30% censoring						
Method	Accuracy	Matthews	NMI	AUC-ROC	Sn	Sp	F1	Accuracy	Matthews	NMI	AUC-ROC	Sn	Sp	F1
**Cox model**	0.67 (0.11)	0.42 (0.19)	0.23 (0.11)	0.66 (0.14)	0.42 (0.05)	0.58 (0.05)	0.43 (0.05)	0.54 (0.11)	0.34 (0.18)	0.11 (0.11)	0.56 (0.1)	0.42 (0.05)	0.58 (0.05)	0.43 (0.05)
**Kernel Cox**	0.74 (0.07)	0.47 (0.13)	0.20 (0.12)	0.78 (0.07)	0.35 (0.13)	0.85 (0.02)	0.48 (0.13)	0.72 (0.08)	0.46 (0.15)	0.17 (0.12)	0.77 (0.09)	0.33 (0.13)	0.85 (0.02)	0.47 (0.13)
**wSVM-KM**	0.62 (0.04)	0.21 (0.16)	0.05 (0.10)	0.77 (0.08)	0.31 (0.13)	0.91 (0.02)	0.48 (0.13)	0.54 (0.05)	0.16 (0.10)	0.01 (0.02)	0.76 (0.09)	0.31 (0.13)	0.91 (0.02)	0.48 (0.13)
**wSVM-Prop**	0.61 (0.04)	0.21 (0.16)	0.05 (0.10)	0.77 (0.08)	0.32 (0.01)	0.92 (0.02)	0.48 (0.14)	0.53 (0.05)	0.15 (0.11)	0.01 (0.02)	0.75 (0.08)	0.32 (0.01)	0.92 (0.02)	0.48 (0.14)
**pSVM-linear-KM**	0.77 (0.09)	0.54 (0.17)	0.26 (0.14)	0.86 (0.08)	0.77 (0.04)	0.85 (0.06)	0.77 (0.05)	0.75 (0.07)	0.50 (0.15)	0.22 (0.12)	0.83 (0.07)	0.76 (0.04)	0.85 (0.06)	0.75 (0.05)
**pSVM-linear-prop**	0.75 (0.07)	0.50 (0.14)	0.25 (0.12)	0.84 (0.07)	0.75 (0.04)	0.83 (0.06)	0.76 (0.05)	0.75 (0.07)	0.49 (0.15)	0.21 (0.13)	0.83 (0.07)	0.72 (0.04)	0.81 (0.06)	0.73 (0.05)
**pSVM-radial-KM**	0.65 (0.05)	0.26 (0.16)	0.07 (0.09)	0.77 (0.07)	0.65 (0.02)	0.87 (0.04)	0.71 (0.17)	0.66 (0.07)	0.33 (0.16)	0.36 (0.27)	0.77 (0.08)	0.65 (0.03)	0.87 (0.04)	0.71 (0.17)
**pSVM-radial-prop**	0.64 (0.05)	0.23 (0.17)	0.06 (0.10)	0.77 (0.07)	0.61 (0.02)	0.85 (0.05)	0.68 (0.14)	0.64 (0.07)	0.31 (0.16)	0.29 (0.34)	0.77 (0.08)	0.61 (0.02)	0.83 (0.05)	0.65 (0.14)
**LUPI-linear-KM**	0.70 (0.08)	0.42 (0.13)	0.26 (0.15)	0.76 (0.08)	0.81 (0.04)	0.72 (0.07)	0.75 (0.04)	0.71 (0.08)	0.40 (0.16)	0.18 (0.12)	0.74 (0.09)	0.81 (0.04)	0.72 (0.07)	0.74 (0.04)
**LUPI-linear-prop**	0.70 (0.08)	0.42 (0.13)	0.26 (0.15)	0.76 (0.08)	0.81 (0.04)	0.72 (0.07)	0.75 (0.04)	0.70 (0.08)	0.40 (0.16)	0.18 (0.12)	0.74 (0.09)	0.81 (0.04)	0.70 (0.07)	0.72 (0.04)
**inSVM-gradient**	0.76 (0.07)	0.52 (0.15)	0.23 (0.13)	0.84 (0.07)	0.87 (0.03)	0.83 (0.06)	0.83 (0.04)	0.74 (0.07)	0.47 (0.14)	0.20 (0.11)	0.82 (0.07)	0.87 (0.03)	0.83 (0.05)	0.83 (0.04)
**inSVM-averaging**	0.77 (0.07)	0.52 (0.15)	0.24 (0.13)	0.85 (0.07)	0.87 (0.03)	0.83 (0.06)	0.83 (0.04)	0.75 (0.06)	0.49 (0.13)	0.21 (0.11)	0.83 (0.07)	0.87 (0.03)	0.83 (0.02)	0.81 (0.04)

Performance of all approaches was worse under non-proportional hazards (Tables 3 and 4). The largest difference between proportionality compared to non-proportionality was in the 300 observations scenario (Table 3) compared to the 50 observations scenario (Table 4).

**Table 3:** Accuracy results in a 300 observations non-proportional hazards, zero skew, 10 and 30% censoring. Prediction accuracy of all tested approaches when simulated data was generated with 300 observations and the following assumptions: non-proportional hazards, zero skew, 10 and 30% censoring. The table summarizes the mean (and standard deviation) of the following metrics: accuracy, Matthews’ correlation, normalized mutual information (NMI), area under the Receiver Operating Characteristic curve (AUC-ROC), sensitivity (Sn), specificity (Sp) and F1-score (F1)

	10% censoring							30% censoring						
Method	Accuracy	Matthews	NMI	AUC-ROC	Sn	Sp	F1	Accuracy	Matthews	NMI	AUC-ROC	Sn	Sp	F1
**Cox** **model**	0.71 (0.02)	0.39 (0.05)	0.10 (0.03)	0.77 (0.03)	0.35 (0.03)	0.69 (0.02)	0.4 (0.04)	0.70 (0.03)	0.39 (0.06)	0.10 (0.04)	0.77 (0.03)	0.35 (0.03)	0.66 (0.02)	0.4 (0.04)
**Kernel Cox**	0.67 (0.02)	0.33 (0.05)	0.10 (0.04)	0.71 (0.03)	0.25 (0.05)	0.88 (0.02)	0.30 (0.08)	0.67 (0.03)	0.32 (0.06)	0.08 (0.04)	0.70 (0.03)	0.22 (0.05)	0.83 (0.02)	0.29 (0.08)
**wSVM-KM**	0.65 (0.02)	0.24 (0.05)	0.01 (0.02)	0.71 (0.03)	0.16 (0.05)	0.94 (0.02)	0.26 (0.08)	0.61 (0.02)	0.16 (0.06)	0.01 (0.02)	0.71 (0.03)	0.16 (0.05)	0.94 (0.02)	0.26 (0.08)
**wSVM-Prop**	0.64 (0.02)	0.24 (0.05)	0.01 (0.02)	0.70 (0.03)	0.16 (0.06)	0.94 (0.02)	0.26 (0.08)	0.61 (0.02)	0.17 (0.07)	0.01 (0.02)	0.70 (0.03)	0.13 (0.06)	0.92 (0.02)	0.22 (0.08)
**pSVM-linear-KM**	0.72 (0.03)	0.39 (0.05)	0.13 (0.04)	0.77 (0.03)	0.65 (0.03)	0.69 (0.03)	0.63 (0.03)	0.69 (0.03)	0.37 (0.05)	0.13 (0.03)	0.75 (0.03)	0.60 (0.03)	0.69 (0.03)	0.60 (0.03)
**pSVM-linear-prop**	0.70 (0.03)	0.38 (0.05)	0.13 (0.03)	0.76 (0.03)	0.60 (0.03)	0.66 (0.04)	0.60 (0.03)	0.69 (0.03)	0.37 (0.05)	0.13 (0.03)	0.75 (0.03)	0.62 (0.03)	0.66 (0.04)	0.60 (0.03)
**pSVM-radial-KM**	0.66 (0.02)	0.28 (0.05)	0.03 (0.03)	0.70 (0.03)	0.50 (0.01)	0.78 (0.09)	0.53 (0.09)	0.66 (0.03)	0.31 (0.07)	0.10 (0.04)	0.70 (0.03)	0.50 (0.01)	0.78 (0.09)	0.53 (0.09)
**pSVM-radial-prop**	0.66 (0.02)	0.28 (0.05)	0.03 (0.03)	0.70 (0.03)	0.46 (0.02)	0.75 (0.01)	0.50 (0.09)	0.66 (0.03)	0.31 (0.07)	0.08 (0.05)	0.70 (0.03)	0.44 (0.02)	0.75 (0.01)	0.50 (0.09)
**LUPI-linear-KM**	0.65 (0.02)	0.27 (0.05)	0.03 (0.03)	0.70 (0.03)	0.61 (0.08)	0.66 (0.06)	0.60 (0.04)	0.65 (0.03)	0.31 (0.05)	0.13 (0.05)	0.70 (0.03)	0.61 (0.08)	0.66 (0.06)	0.60 (0.04)
**LUPI-linear-prop**	0.65 (0.02)	0.27 (0.05)	0.03 (0.03)	0.70 (0.03)	0.61 (0.08)	0.65 (0.06)	0.59 (0.04)	0.65 (0.03)	0.31 (0.05)	0.13 (0.05)	0.70 (0.03)	0.60 (0.08)	0.65 (0.06)	0.58 (0.04)
**inSVM-gradient**	0.70 (0.02)	0.38 (0.05)	0.11 (0.03)	0.76 (0.02)	0.68 (0.04)	0.69 (0.03)	0.67 (0.03)	0.67 (0.03)	0.33 (0.06)	0.11 (0.03)	0.72 (0.03)	0.67 (0.04)	0.69 (0.03)	0.67 (0.03)
**inSVM-averaging**	0.70 (0.02)	0.38 (0.05)	0.11 (0.03)	0.76 (0.02)	0.69 (0.04)	0.69 (0.03)	0.65 (0.03)	0.69 (0.03)	0.37 (0.05)	0.13 (0.03)	0.76 (0.03)	0.69 (0.04)	0.68 (0.03)	0.65 (0.03)

**Table 4:** Accuracy results in a 50 observations non-proportional hazards, zero skew, 10 and 30% censoring. Prediction accuracy of all tested approaches when simulated data was generated with 50 observations and the following assumptions: non-proportional hazards, zero skew, 10 and 30% censoring. The table summarizes the mean (and standard deviation) of the following metrics: accuracy, Matthews’ correlation, normalized mutual information (NMI), area under the Receiver Operating Characteristic curve (AUC-ROC), sensitivity (Sn), specificity (Sp) and F1-score (F1)

	10% censoring							30% censoring						
Method	Accuracy	Matthews	NMI	AUC-ROC	Sn	Sp	F1	Accuracy	Matthews	NMI	AUC-ROC	Sn	Sp	F1
**Cox** **model**	0.59 (0.05)	0.14 (0.15)	0.04 (0.07)	0.55 (0.07)	0.39 (0.05)	0.51 (0.05)	0.40 (0.05)	0.58 (0.06)	0.11 (0.19)	0.07 (0.10)	0.53 (0.07)	0.39 (0.05)	0.51 (0.05)	0.41 (0.04)
**Kernel Cox**	0.61 (0.08)	0.22 (0.15)	0.15 (0.17)	0.64 (0.07)	0.31 (0.13)	0.79 (0.02)	0.46 (0.13)	0.63 (0.07)	0.24 (0.15)	0.07 (0.09)	0.64 (0.08)	0.31 (0.13)	0.79 (0.02)	0.46 (0.13)
**wSVM-KM**	0.62 (0.04)	0.08 (0.14)	0.02 (0.03)	0.64 (0.07)	0.29 (0.13)	0.89 (0.02)	0.43 (0.13)	0.59 (0.03)	0.05 (0.15)	0.02 (0.01)	0.64 (0.08)	0.29 (0.13)	0.89 (0.02)	0.43 (0.13)
**wSVM-Prop**	0.60 (0.04)	0.09 (0.14)	0.02 (0.03)	0.64 (0.07)	0.27 (0.01)	0.82 (0.02)	0.42 (0.14)	0.59 (0.03)	0.05 (0.15)	0.02 (0.01)	0.64 (0.08)	0.27 (0.01)	0.82 (0.02)	0.42 (0.14)
**pSVM-linear-KM**	0.63 (0.07)	0.23 (0.14)	0.08 (0.08)	0.66 (0.09)	0.72 (0.04)	0.80 (0.06)	0.77 (0.05)	0.61 (0.08)	0.22 (0.17)	0.11 (0.09)	0.66 (0.09)	0.72 (0.03)	0.80 (0.06)	0.78 (0.05)
**pSVM-linear-prop**	0.61 (0.07)	0.21 (0.14)	0.07 (0.07)	0.65 (0.09)	0.71 (0.04)	0.75 (0.06)	0.71 (0.05)	0.59 (0.09)	0.17 (0.18)	0.11 (0.09)	0.63 (0.09)	0.71 (0.04)	0.74 (0.06)	0.71 (0.05)
**pSVM-radial-KM**	0.63 (0.04)	0.14 (0.14)	0.04 (0.09)	0.63 (0.08)	0.62 (0.02)	0.82 (0.04)	0.69 (0.17)	0.61 (0.06)	0.17 (0.16)	0.10 (0.21)	0.63 (0.09)	0.62 (0.02)	0.82 (0.04)	0.69 (0.17)
**pSVM-radial-prop**	0.61 (0.04)	0.14 (0.14)	0.10 (0.21)	0.63 (0.08)	0.59 (0.02)	0.79 (0.05)	0.61 (0.14)	0.56 (0.07)	0.12 (0.14)	0.31 (0.36)	0.63 (0.09)	0.59 (0.03)	0.79 (0.03)	0.61 (0.14)
**LUPI-linear-KM**	0.62 (0.07)	0.22 (0.15)	0.07 (0.08)	0.63 (0.08)	0.79 (0.04)	0.69 (0.07)	0.71 (0.04)	0.62 (0.07)	0.18 (0.16)	0.03 (0.07)	0.63 (0.09)	0.79 (0.04)	0.69 (0.07)	0.71 (0.04)
**LUPI-linear-prop**	0.61 (0.07)	0.20 (0.15)	0.07 (0.08)	0.63 (0.08)	0.75 (0.04)	0.62 (0.07)	0.64 (0.04)	0.62 (0.07)	0.18 (0.16)	0.03 (0.07)	0.63 (0.09)	0.75 (0.04)	0.62 (0.06)	0.65 (0.04)
**inSVM-gradient**	0.66 (0.07)	0.27 (0.14)	0.06 (0.07)	0.67 (0.08)	0.83 (0.03)	0.81 (0.06)	0.81 (0.04)	0.64 (0.07)	0.27 (0.15)	0.10 (0.10)	0.69 (0.09)	0.83 (0.03)	0.81 (0.06)	0.81 (0.04)
**inSVM-averaging**	0.66 (0.07)	0.28 (0.14)	0.07 (0.07)	0.67 (0.09)	0.81 (0.03)	0.83 (0.06)	0.82 (0.04)	0.65 (0.07)	0.28 (0.16)	0.11 (0.11)	0.68 (0.09)	0.81 (0.03)	0.83 (0.06)	0.83 (0.04)

In all scenarios, approaches based on conditional survival performed better than those based on proportional follow-up time, particularly when the sample size was 50 observations and especially when hazards were non-proportional. Overall differences between both methods were small (around 0.02 units in accuracy and around 0.02 units in AUC-ROC) but consistent.

The inSVM, based on both gradient and averaging approach, performed closest to the best method within each scenario. Although the averaging approach was slightly better and more insensitive to the proportion of censored observations, there were no clear differences between the averaging and gradient approach.

Other scenarios yielded comparable results and are presented in supplementary Tables S1, S2, S3, S4, S5, S6, S7 and S8.

### Real-life datasets

In the three compared datasets the conditional survival approach attained the largest predictive accuracy based on accuracy values and AUC-ROC (Table 5) when compared with the proportionality approach within each method. Within each dataset the performance of the LUPI method was one of the best, with almost no difference between the conditional survival and the proportionality approach.

**Table 5:** Real-life datasets metrics. A 5-fold nested-cross validation approach is applied into real-life datasets. Mean (standard deviation) of 10 resampling datasets is shown. The table summarizes the mean (and standard deviation) of the following metrics: accuracy, area under the Receiver Operating Characteristic curve (AUC-ROC), sensitivity (Sn), specificity (Sp) and F1-score (F1)

	Lung					Stanford2					PBC				
Method	Accuracy	AUC-ROC	Sn	Sp	F1	Accuracy	AUC-ROC	Sn	Sp	F1	Accuracy	AUC-ROC	Sn	Sp	F1
Cox model	0.61 (0.08)	0.60 (0.08)	0.52 (0.07)	0.39 (0.07)	0.50 (0.08)	0.61 (0.07)	0.61 (0.11)	0.48 (0.07)	0.42 (0.09)	0.51 (0.09)	0.75 (0.11)	0.85 (0.11)	0.58 (0.07)	0.62 (0.09)	0.68 (0.09)
Kernel Cox	0.66 (0.14)	0.52 (0.03)	0.60 (0.10)	0.25 (0.07)	0.49 (0.13)	0.51 (0.21)	0.50 (0.01)	0.56 (0.12)	0.31 (0.07)	0.45 (0.08)	0.55 (0.11)	0.50 (0.08)	0.52 (0.12)	0.30 (0.07)	0.55 (0.08)
wSVM-KM	0.73 (0.08)	0.68 (0.16)	0.63 (0.10)	0.34 (0.07)	0.59 (0.10)	0.59 (0.12)	0.62 (0.04)	0.57 (0.11)	0.29 (0.07)	0.52 (0.10)	0.70 (0.09)	0.75 (0.10)	0.67 (0.04)	0.65 (0.11)	0.39 (0.07)
wSVM-Prop	0.70 (0.12)	0.64 (0.15)	0.62 (0.12)	0.33 (0.07)	0.59 (0.12)	0.55 (0.09)	0.59 (0.10)	0.56 (0.12)	0.27 (0.07)	0.51 (0.12)	0.70 (0.07)	0.73 (0.05)	0.59 (0.10)	0.60 (0.12)	0.41 (0.09)
pSVM-linear-KM	0.89 (0.08)	0.72 (0.16)	0.63 (0.10)	0.23 (0.11)	0.62 (0.11)	0.78 (0.07)	0.63 (0.11)	0.57 (0.10)	0.21 (0.11)	0.58 (0.11)	0.67 (0.08)	0.84 (0.08)	0.65 (0.09)	0.61 (0.09)	0.35 (0.12)
pSVM-linear-prop	0.88 (0.08)	0.70 (0.15)	0.65 (0.16)	0.36 (0.13)	0.61 (0.11)	0.77 (0.07)	0.63 (0.10)	0.56 (0.16)	0.24 (0.13)	0.57 (0.11)	0.65 (0.12)	0.80 (0.10)	0.68 (0.13)	0.45 (0.11)	0.26 (0.13)
pSVM-radial-KM	0.82 (0.08)	0.70 (0.14)	0.69 (0.09)	0.55 (0.09)	0.63 (0.09)	0.60 (0.10)	0.61 (0.08)	0.43 (0.09)	0.45 (0.12)	0.50 (0.09)	0.70 (0.07)	0.65 (0.13)	0.65 (0.08)	0.50 (0.09)	0.55 (0.12)
pSVM-radial-prop	0.81 (0.08)	0.69 (0.15)	0.61 (0.11)	0.55 (0.07)	0.62 (0.10)	0.60 (0.11)	0.59 (0.10)	0.44 (0.06)	0.51 (0.11)	0.52 (0.10)	0.70 (0.07)	0.61 (0.10)	0.63 (0.10)	0.44 (0.06)	0.51 (0.07)
LUPI-linear-KM	0.92 (0.05)	0.65 (0.14)	0.72 (0.14)	0.67 (0.10)	0.69 (0.11)	0.80 (0.07)	0.63 (0.12)	0.65 (0.11)	0.51 (0.10)	0.61 (0.11)	0.70 (0.07)	0.63 (0.09)	0.63 (0.12)	0.55 (0.11)	0.50 (0.10)
LUPI-linear-prop	0.92 (0.05)	0.65 (0.14)	0.71 (0.11)	0.61 (0.12)	0.67 (0.09)	0.80 (0.07)	0.63 (0.12)	0.65 (0.10)	0.53 (0.13)	0.58 (0.09)	0.70 (0.07)	0.63 (0.09)	0.61 (0.12)	0.57 (0.13)	0.53 (0.13)
inSVM-gradient	0.67 (0.08)	0.68 (0.08)	0.60 (0.10)	0.43 (0.12)	0.58 (0.08)	0.52 (0.11)	0.59 (0.10)	0.49 (0.10)	0.43 (0.12)	0.58 (0.08)	0.68 (0.10)	0.60 (0.15)	0.59 (0.11)	0.52 (0.12)	0.46 (0.12)
inSVM-averaging	0.85 (0.07)	0.71 (0.13)	0.76 (0.13)	0.43 (0.07)	0.65 (0.12)	0.78 (0.06)	0.67 (0.13)	0.69 (0.13)	0.56 (0.12)	0.56 (0.09)	0.75 (0.02)	0.74 (0.10)	0.68 (0.11)	0.61 (0.12)	0.57 (0.13)

The inSVM method averaging approach performed better than gradient in both accuracy and AUC-ROC metrics in all three datasets, being the former one of the best methods within each ones of the datasets.

## Discussion and conclusions

In this article we proposed alternative methods and extensions within the SVM for binary classification framework for dealing with censored data. Specifically, a conditional survival approach for weighting censored observations when fitting SVM through LUPI, Uncertainty SVM, Weighted SVM, and a semi-supervised SVM with local invariances. The former takes into account the events and follow-up period including more information in the weighting process than using a proportionality of time approach. The latter is a semi-supervised SVM with local invariances method that allows using two types of invariances: gradient over variables and averaging over observations. We showed that both approaches outperformed the other studied methods on most compared metrics.

As expected, when the sample size was as limited as 50 observations and the proportional hazards assumption was violated, the Cox proportional hazards model had a poorer performance. Results with the wSVM, were highly dependent on the proportion of censoring but not so much on the distribution of time to censor. Moreover, wSVM results were comparable to the LUPI results, and that has also been observed by Lapin et al. [24]. This similarity may be explained by the common unique information (censored data) used by both methods. This similarity suggests that the wSVM method may be more advantageous in practice because is much less time consuming, although is less robust than the LUPI method.

When applying the LUPI approach, we have included the censoring data as privileged information in the correcting space. Our results were consistent with Shiao and Cherkassky [15], i.e., LUPI performs worse than the Cox proportional hazards model and pSVM in all compared scenarios. Actually, some of our simulated scenarios were similar to simulated scenarios used by Shiao and Cherkassky. The correcting space is used as complementary information to be combined with the decision space. Therefore, is not directly used to define the class of the observations, as it is in pSVM or wSVM. We agree with Serra-Toro et al. [25], that further work is needed to fully understand the LUPI approach and how the correcting and decision spaces interact.

The performance of the pSVM and the Cox proportional hazards model was similar when the sample size was larger and better than the kernel Cox regression, being the linear kernel slightly superior to the radial kernel, as observed by Shiao and Cherkassky [15]. Perhaps a finer grid search could benefit the overall performance of the non-linear approach. Ours and Shiao and Cherkassky [15] results were consistent with regards to the superior performance of the linear pSVM performs when compared to the pSVM using Gaussian kernel.

The conditional survival approach proposed by us performs better than the proportional follow-up time approach in all compared scenarios. The conditional method takes into account the events and follow-up period, hereby, it includes more information and is more accurate in the weighting estimation than the proportionality of time approach. The latter is assuming linearity and does not take into account specificities of the data, for instance, variability in survival due to intrinsic data. However, one aspect to be remarked is that the conditional approach is assuming that the survival probability of the test data is similar to the training data. This is a reasonable assumption but depending on the difference between survival probabilities, the prediction accuracy may be affected.

With respect to the proposed inSVM approach (both gradient and averaging), in the 300 observations scenarios, results are pretty similar to Cox, kernel Cox regression and pSVM. However, in scenarios in which the number of observations was small and close to the number of variables, the inSVM outperformed all other approaches in all compared metrics, and it was one of the most robust approaches to varying number of variables and violations of proportionality of hazards. Although, inSVM is a semi-supervised approach that does not account for censoring, its performance is comparable to other methods that account for censoring. That could be explained because we are assuming that censoring is independent from the events and representative of the data. Therefore, patterns in the observed data that are applicable to the censored observations and the local invariances assumptions should be valid. Additionally, an advantage of this approach is that no extra assumptions about the censoring distribution are necessary. The main drawback of the local invariances approach is that it is computationally intensive, specially the gradient approach.

All simulated data was based on balanced data, i.e., the proportion of events and non-events were similar. SVM models are sensitive to data imbalance between classes. Therefore, future investigation shall consider imbalanced scenarios.

Given the significant number of compared methods and data, the presented work has been restricted to the two most commonly used linear and Gaussian kernels. Further work shall evaluate the performance of the proposed methods using other kernels. Additionally, we addressed overfitting through standard procedures: by simulating completely different datasets to test parameters and validate models, and by applying nested cross-validation to estimate and validate parameters when analysing real data. However, future work may assess the performance of the proposed methods including even more simulation scenarios and a larger range of parameter values.

From the compared methods the proposed inSVM method using the conditional survival approach is the most robust under different scenarios and is a good approach to consider as an alternative to other time-to-event methods. When analysing sparse data is a method to be considered and recommended since outperforms other methods even when the proportional hazards assumption is not met, a situation that often occurs in biomedical data and biomarkers analysis.

## Supplementary information

**Additional file 1: Table S1. Proportional hazards, positive skew, 10 and 30% censoring and 300 observations scenarios results.** Mean (standard deviation) of accuracy, Matthews’ correlation, normalized mutual information (NMI), area under the ROC curve (AUC), sensitivity (Sn), specificity (Sp) and F1-score (F1) is shown.

**Additional file 2: Table S2. Proportional hazards, negative skew, 10 and 30% censoring and 300 observations scenarios results.** Mean (standard deviation) of accuracy, Matthews’ correlation, normalized mutual information (NMI), area under the ROC curve (AUC), sensitivity (Sn), specificity (Sp) and F1-score (F1) is shown.

**Additional file 3: Table S3. Non-proportional hazards, negative skew, 10 and 30% censoring and 300 observations scenarios results.** Mean (standard deviation) of accuracy, Matthews’ correlation, normalized mutual information (NMI), area under the ROC curve (AUC), sensitivity (Sn), specificity (Sp) and F1-score (F1) is shown.

**Additional file 4: Table S4. Non-proportional hazards, positive skew, 10 and 30% censoring and 300 observations scenarios results.** Mean (standard deviation) of accuracy, Matthews’ correlation, normalized mutual information (NMI), area under the ROC curve (AUC), sensitivity (Sn), specificity (Sp) and F1-score (F1) is shown.

**Additional file 5: Table S5. Proportional hazards, negative skew, 10 and 30% censoring and 50 observations scenarios results.** Mean (standard deviation) of accuracy, Matthews’ correlation, normalized mutual information (NMI), area under the ROC curve (AUC), sensitivity (Sn), specificity (Sp) and F1-score (F1) is shown.

**Additional file 6: Table S6. Proportional hazards, positive skew, 10 and 30% censoring and 50 observations scenarios results.** Mean (standard deviation) of accuracy, Matthews’ correlation, normalized mutual information (NMI), area under the ROC curve (AUC), sensitivity (Sn), specificity (Sp) and F1-score (F1) is shown.

**Additional file 7: Table S7. Non-proportional hazards, negative skew, 10 and 30% censoring and 50 observations scenarios results.** Mean (standard deviation) of accuracy, Matthews’ correlation, normalized mutual information (NMI), area under the ROC curve (AUC), sensitivity (Sn), specificity (Sp) and F1-score (F1) is shown.

**Additional file 8: Table S8. Non-proportional hazards, positive skew, 10 and 30% censoring and 50 observations scenarios results.** Mean (standard deviation) of accuracy, Matthews’ correlation, normalized mutual information (NMI), area under the ROC curve (AUC), sensitivity (Sn), specificity (Sp) and F1-score (F1) is shown.

## Data Availability

Simulated datasets during the current study are available from the corresponding author on reasonable request. PBC, Lung and Stanford datasets are freely available at https://CRAN.R-project.org/package=survival.

## Abbreviations

SVM: Support vector machines
SVR: Support vector regression
LUPI: Learning using privileged information
inSVM: Invariances support vector machine
pSVM: Probabilistic support vector machine
wSVM: Weighted support vector machines
