# Correction to: Enhancing SVM for survival data using local invariances and weighting

**DOI:** 10.1186/s12859-020-03558-7

**Published:** 2020-08-27

**Authors:** Hector Sanz, Ferran Reverter, Clarissa Valim

**Affiliations:** 1 Department of Genetics, Microbiology and Statistics, Faculty of Biology, Universitat de Barcelona, Diagonal, 643, 08028 Barcelona, Catalonia Spain; 2 Centre for Genomic Regulation (CRG), The Barcelona Institute for Science and Technology, Dr. Aiguader 88, 08003 Barcelona, Spain; 3 Department of Global Health, Boston University, 801 Massachusetts Avenue, Boston, MA 02118 USA; 4 Department of Immunology and Infectious Diseases, Harvard T.H. Chen School of Public Health, 675 Huntington Ave, Boston, MA 02115 USA

**Correction to: BMC Bioinformatics 21, 193 (2020)**

**https://doi.org/10.1186/s12859-020-3481-2**

Following publication of the original article [1], the authors identified errors in the equations. The correct equations are given below.

Equation (7)

$$ {S}^{z(ti)}=\frac{S^{\left( ti+z\right)}}{S^{(ti)}} $$

Equation (8)

$$ {\displaystyle \begin{array}{l}\underset{\boldsymbol{w},{\boldsymbol{w}}^{\ast },b,{b}^{\ast }}{\operatorname{minimize}}\kern1em \frac{1}{2}\left({\left\Vert \boldsymbol{w}\right\Vert}^2+\gamma {\left\Vert {\boldsymbol{w}}^{\ast}\right\Vert}^2\right)+C\sum \limits_{i=1}^n{\xi}_i\\ {}\mathrm{subject}\kern0.5em \mathrm{to}\kern0.5em {\xi}_i=\left(\left\langle {\boldsymbol{w}}^{\ast },{\boldsymbol{x}}_i^{\ast}\right\rangle +{b}^{\ast}\right),\kern9em i=1,\dots, n\\ {}\kern5em {y}_i\left(\left\langle \boldsymbol{w},{\boldsymbol{x}}_i\right\rangle +b\right)\ge 1-\left(\left\langle {\boldsymbol{w}}^{\ast },{\boldsymbol{x}}_i^{\ast}\right\rangle +{b}^{\ast}\right),\kern3.2em i=1,\dots, n\\ {}\kern5em \left(\left\langle {\boldsymbol{w}}^{\ast },{\boldsymbol{x}}_i^{\ast}\right\rangle +{b}^{\ast}\right)\ge 0,\kern9.6em i=1,\dots, n\end{array}} $$

Equation (10)

$$ {\displaystyle \begin{array}{l}\underset{\boldsymbol{w},b}{\operatorname{minimize}}\kern1em \frac{1}{2}{\left\Vert \boldsymbol{w}\right\Vert}^2\\ {}\mathrm{subject}\kern0.5em \mathrm{to}\kern0.5em {y}_i\left(\left\langle \boldsymbol{w},{\boldsymbol{x}}_i\right\rangle =b\right)\ge 1,\kern4em i=1,\dots, n\\ {}\kern4em {z}_i^{-}\le \left\langle \boldsymbol{w},{\boldsymbol{x}}_i\right\rangle +b\le {z}_i^{+},\kern3.5em i=n+1,\dots, m\end{array}} $$

Re-expression of Equation (10)

$$ {\displaystyle \begin{array}{l}\underset{\boldsymbol{w},\xi, {\xi}^{-},{\xi}^{+},b}{\operatorname{minimize}}\kern1em \frac{1}{2}{\left\Vert \boldsymbol{w}\right\Vert}^2+C\sum \limits_{i=1}^n{\xi}_i+\tilde{C}\sum \limits_{i=n+1}^m\left({\xi}_i^{-}+{\xi}_i^{+}\right)\\ {}\mathrm{subject}\kern0.5em \mathrm{to}\kern0.5em {y}_i\left(\left\langle \boldsymbol{w},{\boldsymbol{x}}_i\right\rangle +b\right)\ge 1-{\xi}_i,\kern5em i=1,\dots, n\\ {}\kern5em {z}_i^{-}-{\xi}_i^{-}\le \left\langle \boldsymbol{w},{\boldsymbol{x}}_i\right\rangle +b\le {z}_i^{+}+{\xi}_i^{+},\kern1.8em i=n+1,\dots, m\\ {}\kern5em {\xi}_i\ge 0,\kern11.5em i=1,\dots, n\\ {}\kern5em {\xi}_i^{-}\ge 0,\kern11.2em i=n+1,\dots, m\\ {}\kern5em {\xi}_i^{+}\ge 0,\kern11em i=n+1,\dots, m\end{array}} $$

Equation (11)

$$ {\displaystyle \begin{array}{l}\underset{\boldsymbol{w},b}{\operatorname{minimize}}\kern1em \frac{1}{2}{\left\Vert \boldsymbol{w}\right\Vert}^2\\ {}\mathrm{subject}\kern0.5em \mathrm{to}\kern0.5em {y}_i\left(\left\langle \boldsymbol{w},{\boldsymbol{x}}_i\right\rangle +b\right)\ge 1,\kern7em i=1,\dots, n\\ {}\kern5em {z}_i^{-}\le \left\langle \boldsymbol{w},{\boldsymbol{x}}_i\right\rangle +b\le {z}_i^{+},\kern6.3em i=n+1,\dots, m\end{array}} $$

Equation (13)

$$ {\displaystyle \begin{array}{l}\underset{\boldsymbol{w},\boldsymbol{\xi}}{\operatorname{minimize}}\kern1em \frac{1}{2}{\left\Vert \boldsymbol{w}\right\Vert}^2+C\sum \limits_{i=1}^n{W}_i{\xi}_i\\ {}\mathrm{subject}\kern0.5em \mathrm{to}\kern0.5em {y}_i\left(\left\langle \boldsymbol{w},{\boldsymbol{x}}_i\right\rangle +b\right)\ge 1-{\xi}_i,\kern7em i=1,\dots, n\\ {}\kern5em {\xi}_i\ge 0,\kern13.5em i=1,\dots, n\end{array}} $$

Equation (16)

$$ {z}_{x_{i,j}}\left(\boldsymbol{x}\right)=\frac{1}{\sigma^2}\left({x}^j-{x}_i^j\right)\mathit{\exp}\left(-\frac{1}{2{\sigma}^2}{\left\Vert \boldsymbol{x}={\boldsymbol{x}}_i\right\Vert}^2\right) $$

The original article has been corrected.
