# Supplementary material for: Enhancing SVM for survival data using local invariances and weighting
Source: BMC Bioinformatics. 2020 May 19;21:193. doi: 10.1186/s12859-020-3481-2 (PMC7236493; doi:10.1186/s12859-020-3481-2)
Supplement: Supplementary file 1 — Additional file 1: Table S1. Proportional hazards, positive skew, 10 and 30% censoring and 300 observations scenarios results. Mean (standard deviation) of accuracy, Matthews’ correlation, normalized mutual information (NMI), area under the ROC curve (AUC), sensitivity (Sn), specificity (Sp) and F1-score (F1) is shown. [file 12859_2020_3481_MOESM1_ESM.docx]

|  | **10% censoring** | | | | | | | **30% censoring** | | | | | | |
| --- | --- | --- | --- | --- | --- | --- | --- | --- | --- | --- | --- | --- | --- | --- |
| **Method** | **Accuracy** | **Matthews** | **NMI** | **AUC-ROC** | **Sn** | **Sp** | **F1** | **Accuracy** | **Matthews** | **NMI** | **AUC-ROC** | **Sn** | **Sp** | **F1** |
| **Cox**  **model** | 0.89  (0.02) | 0.77  (0.03) | 0.49 (0.05) | 0.96 (0.01) | 0.61 (0.04) | 0.60 (0.04) | 0.60 (0.04) | 0.89  (0.02) | 0.78  (0.04) | 0.50 (0.06) | 0.96 (0.01) | 0.61 (0.03) | 0.60 (0.04) | 0.60 (0.04) |
| **Kernel Cox** | 0.80  (0.03) | 0.59  (0.06) | 0.27 (0.06) | 0.86 (0.03) | 0.42 (0.01) | 0.91 (0.03) | 0.50 (0.01) | 0.79  (0.02) | 0.58  (0.05) | 0.25 (0.05) | 0.86 (0.02) | 0.32 (0.01) | 0.93 (0.03) | 0.52 (0.01) |
| **wSVM-KM** | 0.75  (0.02) | 0.45  (0.06) | 0.15 (0.04) | 0.87 (0.02) | 0.40 (0.01) | 0.95 (0.03) | 0.54 (0.01) | 0.67  (0.02) | 0.37  (0.05) | 0.11 (0.03) | 0.85 (0.03) | 0.38 (10.25) | 0.95 (0.03) | 0.55 (0.1) |
| **wSVM-Prop** | 0.73  (0.02) | 0.46  (0.06) | 0.15 (0.04) | 0.87 (0.02) | 0.39  (0.01) | 0.95 (0.03) | 0.53 (0.01) | 0.66  (0.02) | 0.36  (0.04) | 0.10 (0.03) | 0.85 (0.03) | 0.39 (0.01) | 0.92 (0.03) | 0.54 (0.1) |
| **pSVM-linear-KM** | 0.87  (0.02) | 0.72  (0.05) | 0.44 (0.06) | 0.95 (0.02) | 0.85 (0.03) | 0.84 (0.03) | 0.81 (0.02) | 0.86  (0.02) | 0.71  (0.04) | 0.42 (0.06) | 0.94 (0.01) | 0.87 (0.04) | 0.86 (0.03) | 0.82 (0.02) |
| **pSVM-linear-prop** | 0.86  (0.02) | 0.72  (0.05) | 0.44 (0.06) | 0.95 (0.02) | 0.85 (0.04) | 0.85 (0.03) | 0.80 (0.03) | 0.86  (0.02) | 0.71  (0.04) | 0.42 (0.06) | 0.94 (0.01) | 0.86 (0.04) | 0.86 (0.03) | 0.80 (0.03) |
| **pSVM-radial-KM** | 0.79  (0.02) | 0.57  (0.05) | 0.25 (0.05) | 0.87 (0.02) | 0.69 (0.07) | 0.88 (0.04) | 0.73 (0.05) | 0.79  (0.03) | 0.58  (0.05) | 0.27 (0.05) | 0.86 (0.02) | 0.67 (0.07) | 0.88 (0.04) | 0.74 (0.05) |
| **pSVM-radial-prop** | 0.79  (0.02) | 0.56  (0.05) | 0.24 (0.05) | 0.87 (0.02) | 0.67 (0.07) | 0.85 (0.04) | 0.72 (0.05) | 0.79  (0.03) | 0.57  (0.05) | 0.27 (0.05) | 0.86 (0.02) | 0.69 (0.03) | 0.87 (0.05) | 0.74 (0.05) |
| **LUPI-linear-KM** | 0.78  (0.03) | 0.56  (0.05) | 0.27 (0.05) | 0.84 (0.03) | 0.81 (0.04) | 0.74 (0.04) | 0.75 (0.03) | 0.78  (0.03) | 0.54  (0.05) | 0.26 (0.05) | 0.84 (0.03) | 0.81 (0.04) | 0.73 (0.04) | 0.77 (0.03) |
| **LUPI-linear-prop** | 0.77  (0.03) | 0.55  (0.05) | 0.27 (0.05) | 0.84 (0.03) | 0.81 (0.04) | 0.74 (0.04) | 0.73 (0.03) | 0.77  (0.03) | 0.54  (0.05) | 0.26 (0.05) | 0.83 (0.03) | 0.81 (0.04) | 0.73 (0.04) | 0.75 (0.03) |
| **inSVM-gradient** | 0.83  (0.02) | 0.66  (0.04) | 0.35 (0.05) | 0.92 (0.02) | 0.87 (0.04) | 0.90 (0.03) | 0.84 (0.04) | 0.80  (0.02) | 0.59  (0.05) | 0.27 (0.05) | 0.88 (0.02) | 0.87 (0.04) | 0.90 (0.03) | 0.82 (0.04) |
| **inSVM-averaging** | 0.84  (0.02) | 0.67  (0.05) | 0.37 (0.05) | 0.92 (0.02) | 0.88 (0.04) | 0.89 (0.03) | 0.83 (0.04) | 0.83  (0.02) | 0.66  (0.05) | 0.35 (0.06) | 0.92 (0.02) | 0.88 (0.04) | 0.89 (0.03) | 0.84 (0.04) |
