# Supplementary material for: Enhancing SVM for survival data using local invariances and weighting
Source: BMC Bioinformatics. 2020 May 19;21:193. doi: 10.1186/s12859-020-3481-2 (PMC7236493; doi:10.1186/s12859-020-3481-2)
Supplement: Supplementary file 3 — Additional file 3: Table S3. Non-proportional hazards, negative skew, 10 and 30% censoring and 300 observations scenarios results. Mean (standard deviation) of accuracy, Matthews’ correlation, normalized mutual information (NMI), area under the ROC curve (AUC), sensitivity (Sn), specificity (Sp) and F1-score (F1) is shown. [file 12859_2020_3481_MOESM3_ESM.docx]

|  | **10% censoring** | | | | | | | **30% censoring** | | | | | | |
| --- | --- | --- | --- | --- | --- | --- | --- | --- | --- | --- | --- | --- | --- | --- |
| **Method** | **Accuracy** | **Matthews** | **NMI** | **AUC-ROC** | **Sn** | **Sp** | **F1** | **Accuracy** | **Matthews** | **NMI** | **AUC-ROC** | **Sn** | **Sp** | **F1** |
| **Cox**  **model** | 0.71  (0.03) | 0.39  (0.06) | 0.10 (0.04) | 0.77 (0.03) | 0.33 (0.03) | 0.69 (0.02) | 0.4 (0.04) | 0.70  (0.03) | 0.38  (0.05) | 0.09 (0.03) | 0.76 (0.03) | 0.35 (0.03) | 0.66 (0.02) | 0.40 (0.04) |
| **Kernel Cox** | 0.67  (0.03) | 0.33  (0.05) | 0.1 (0.04) | 0.71 (0.03) | 0.25 (0.05) | 0.88 (0.02) | 0.30 (0.08) | 0.68  (0.02) | 0.33  (0.05) | 0.08 (0.04) | 0.7 (0.03) | 0.22 (0.05) | 0.83 (0.02) | 0.28  (0.08) |
| **wSVM-KM** | 0.65  (0.02) | 0.24  (0.06) | 0.01 (0.02) | 0.71 (0.03) | 0.16 (0.05) | 0.94 (0.02) | 0.26 (0.08) | 0.63  (0.02) | 0.19  (0.06) | 0.01 (0.02) | 0.70 (0.03) | 0.16 (0.05) | 0.94 (0.02) | 0.26 (0.08) |
| **wSVM-Prop** | 0.64  (0.02) | 0.24  (0.06) | 0.01 (0.02) | 0.71 (0.03) | 0.16 (0.06) | 0.94 (0.02) | 0.26 (0.08) | 0.62  (0.02) | 0.18  (0.06) | 0.01 (0.02) | 0.69 (0.02) | 0.14 (0.06) | 0.92 (0.02) | 0.22 (0.08) |
| **pSVM-linear-KM** | 0.69  (0.03) | 0.38  (0.06) | 0.13 (0.04) | 0.76 (0.03) | 0.65 (0.03) | 0.69 (0.03) | 0.63 (0.03) | 0.68  (0.03) | 0.36  (0.05) | 0.13 (0.04) | 0.75 (0.03) | 0.60 (0.03) | 0.69 (0.03) | 0.60 (0.03) |
| **pSVM-linear-prop** | 0.69  (0.03) | 0.37  (0.06) | 0.13 (0.04) | 0.76 (0.03) | 0.61 (0.03) | 0.66 (0.04) | 0.60 (0.03) | 0.68  (0.03) | 0.35  (0.06) | 0.12 (0.04) | 0.75 (0.03) | 0.61 (0.03) | 0.66 (0.04) | 0.60 (0.03) |
| **pSVM-radial-KM** | 0.66  (0.02) | 0.29  (0.05) | 0.04 (0.03) | 0.71 (0.03) | 0.50 (0.01) | 0.78 (0.09) | 0.54 (0.09) | 0.65  (0.04) | 0.31  (0.06) | 0.20 (0.11) | 0.71 (0.02) | 0.50 (0.01) | 0.78 (0.09) | 0.52 (0.09) |
| **pSVM-radial-prop** | 0.66  (0.02) | 0.29  (0.05) | 0.03 (0.03) | 0.70 (0.03) | 0.46 (0.02) | 0.75 (0.01) | 0.50 (0.09) | 0.64  (0.04) | 0.30  (0.06) | 0.17 (0.11) | 0.70 (0.02) | 0.44 (0.02) | 0.75 (0.01) | 0.50 (0.09) |
| **LUPI-linear-KM** | 0.66  (0.03) | 0.33  (0.05) | 0.15 (0.06) | 0.70 (0.03) | 0.61 (0.08) | 0.66 (0.06) | 0.59 (0.04) | 0.66  (0.03) | 0.33  (0.05) | 0.13 (0.06) | 0.70 (0.02) | 0.61 (0.08) | 0.66 (0.06) | 0.60 (0.04) |
| **LUPI-linear-prop** | 0.66  (0.03) | 0.33  (0.05) | 0.15 (0.06) | 0.70 (0.03) | 0.61 (0.08) | 0.65 (0.06) | 0.59 (0.04) | 0.66  (0.03) | 0.33  (0.05) | 0.13 (0.06) | 0.70 (0.02) | 0.60 (0.08) | 0.65 (0.06) | 0.58 (0.04) |
| **inSVM-gradient** | 0.69  (0.03) | 0.37  (0.05) | 0.11 (0.03) | 0.76 (0.03) | 0.68 (0.04) | 0.69 (0.03) | 0.67 (0.03) | 0.67  (0.02) | 0.34  (0.05) | 0.13 (0.05) | 0.72 (0.02) | 0.67 (0.04) | 0.69 (0.03) | 0.67 (0.03) |
| **inSVM-averaging** | 0.69  (0.03) | 0.37  (0.05) | 0.12 (0.03) | 0.76 (0.03) | 0.69 (0.04) | 0.69 (0.03) | 0.62 (0.03) | 0.68  (0.03) | 0.37  (0.05) | 0.14 (0.04) | 0.76 (0.03) | 0.69 (0.04) | 0.68 (0.03) | 0.65 (0.03) |
