# Supplementary material for: Enhancing SVM for survival data using local invariances and weighting
Source: BMC Bioinformatics. 2020 May 19;21:193. doi: 10.1186/s12859-020-3481-2 (PMC7236493; doi:10.1186/s12859-020-3481-2)
Supplement: Supplementary file 6 — Additional file 6: Table S6. Proportional hazards, positive skew, 10 and 30% censoring and 50 observations scenarios results. Mean (standard deviation) of accuracy, Matthews’ correlation, normalized mutual information (NMI), area under the ROC curve (AUC), sensitivity (Sn), specificity (Sp) and F1-score (F1) is shown. [file 12859_2020_3481_MOESM6_ESM.docx]

|  | **10% censoring** | | | | | | | **30% censoring** | | | | | | |
| --- | --- | --- | --- | --- | --- | --- | --- | --- | --- | --- | --- | --- | --- | --- |
| **Method** | **Accuracy** | **Matthews** | **NMI** | **AUC-ROC** | **Sn** | **Sp** | **F1** | **Accuracy** | **Matthews** | **NMI** | **AUC-ROC** | **Sn** | **Sp** | **F1** |
| **Cox**  **model** | 0.67  0.14) | 0.47  (0.18) | 0.24 (0.14) | 0.68 (0.14) | 0.42 (0.05) | 0.58 (0.05) | 0.43 (0.06) | 0.52  (0.10) | 0.28  (0.14) | 0.07 (0.08) | 0.55 (0.09) | 0.42 (0.05) | 0.58 (0.05) | 0.43 (0.05) |
| **Kernel Cox** | 0.74  (0.06) | 0.48  (0.13) | 0.18 (0.1) | 0.79 (0.07) | 0.34 (0.13) | 0.85 (0.02) | 0.48 (0.13) | 0.73  (0.07) | 0.45  (0.16) | 0.17 (0.11) | 0.77 (0.08) | 0.33 (0.13) | 0.85 (0.02) | 0.47 (0.13) |
| **wSVM-KM** | 0.62  (0.06) | 0.28  (0.16) | 0.09 (0.08) | 0.79 (0.07) | 0.31 (0.13) | 0.91 (0.02) | 0.48 (0.13) | 0.54  (0.05) | 0.15  (0.09) | 0.02 (0) | 0.78 (0.07) | 0.31 (0.13) | 0.92 (0.02) | 0.48 (0.13) |
| **wSVM-Prop** | 0.60  (0.06) | 0.27  (0.16) | 0.08 (0.08) | 0.79 (0.07) | 0.32 (0.01) | 0.92 (0.02) | 0.48 (0.14) | 0.54  (0.05) | 0.14  (0.08) | 0.02 (0.01) | 0.77 (0.07) | 0.32 (0.01) | 0.92 (0.02) | 0.48 (0.14) |
| **pSVM-linear-KM** | 0.77  (0.06) | 0.54  (0.12) | 0.26 (0.11) | 0.86 (0.06) | 0.77 (0.04) | 0.84 (0.06) | 0.77 (0.05) | 0.74  (0.07) | 0.47  (0.14) | 0.21 (0.11) | 0.82 (0.07) | 0.76 (0.04) | 0.85 (0.06) | 0.75 (0.05) |
| **pSVM-linear-prop** | 0.77  (0.07) | 0.54  (0.13) | 0.26 (0.12) | 0.85 (0.06) | 0.75 (0.04) | 0.83 (0.06) | 0.76  (0.05) | 0.74  (0.07) | 0.47  (0.14) | 0.21 (0.11) | 0.82 (0.07) | 0.72 (0.04) | 0.81 (0.06) | 0.73  (0.05) |
| **pSVM-radial-KM** | 0.67  (0.06) | 0.33  (0.17) | 0.23 (0.25) | 0.79 (0.07) | 0.65 (0.02) | 0.87 (0.04) | 0.71 (0.17) | 0.64  (0.07) | 0.30  (0.15) | 0.40 (0.41) | 0.76 (0.08) | 0.65 (0.03) | 0.87 (0.04) | 0.71 (0.17) |
| **pSVM-radial-prop** | 0.66  (0.07) | 0.31  (0.17) | 0.22 (0.27) | 0.79 (0.07) | 0.61 (0.02) | 0.85 (0.05) | 0.68 (0.14) | 0.64  (0.08) | 0.29  (0.17) | 0.35 (0.41) | 0.76 (0.08) | 0.61 (0.02) | 0.83 (0.05) | 0.65 (0.13) |
| **LUPI-linear-KM** | 0.73  (0.06) | 0.47  (0.12) | 0.22 (0.1) | 0.79 (0.07) | 0.81 (0.04) | 0.72 (0.07) | 0.75 (0.04) | 0.71  (0.06) | 0.42  (0.13) | 0.21 (0.11) | 0.76 (0.07) | 0.81 (0.04) | 0.73 (0.07) | 0.74 (0.04) |
| **LUPI-linear-prop** | 0.73  (0.06) | 0.47  (0.12) | 0.22 (0.1) | 0.79 (0.07) | 0.81 (0.04) | 0.71 (0.07) | 0.75 (0.04) | 0.71  (0.06) | 0.42  (0.13) | 0.21 (0.11) | 0.76 (0.07) | 0.81 (0.04) | 0.70 (0.07) | 0.72 (0.04) |
| **inSVM-gradient** | 0.76  (0.06) | 0.52  (0.12) | 0.22 (0.10) | 0.85 (0.06) | 0.87 (0.03) | 0.83 (0.06) | 0.83 (0.04) | 0.72  (0.07) | 0.45  (0.14) | 0.22 (0.12) | 0.79 (0.07) | 0.87 (0.03) | 0.83 (0.05) | 0.83 (0.04) |
| **inSVM-averaging** | 0.76  (0.07) | 0.51  (0.14) | 0.22 (0.11) | 0.84 (0.06) | 0.87 (0.03) | 0.83 (0.06) | 0.83 (0.04) | 0.75  (0.06) | 0.50  (0.11) | 0.24 (0.10) | 0.83 (0.06) | 0.87 (0.03) | 0.83 (0.02) | 0.81 (0.04) |
