# Supplementary material for: Enhancing SVM for survival data using local invariances and weighting
Source: BMC Bioinformatics. 2020 May 19;21:193. doi: 10.1186/s12859-020-3481-2 (PMC7236493; doi:10.1186/s12859-020-3481-2)
Supplement: Supplementary file 8 — Additional file 8: Table S8. Non-proportional hazards, positive skew, 10 and 30% censoring and 50 observations scenarios results. Mean (standard deviation) of accuracy, Matthews’ correlation, normalized mutual information (NMI), area under the ROC curve (AUC), sensitivity (Sn), specificity (Sp) and F1-score (F1) is shown. [file 12859_2020_3481_MOESM8_ESM.docx]

|  | **10% censoring** | | | | | | | **30% censoring** | | | | | | |
| --- | --- | --- | --- | --- | --- | --- | --- | --- | --- | --- | --- | --- | --- | --- |
| **Method** | **Accuracy** | **Matthews** | **NMI** | **AUC-ROC** | **Sn** | **Sp** | **F1** | **Accuracy** | **Matthews** | **NMI** | **AUC-ROC** | **Sn** | **Sp** | **F1** |
| **Cox**  **model** | 0.56  (0.04) | 0.08  (0.11) | 0.02 (0.07) | 0.52 (0.05) | 0.39 (0.05) | 0.51 (0.05) | 0.40 (0.05) | 0.57  (0.05) | 0.12  (0.13) | 0.07 (0.08) | 0.53 (0.06) | 0.39 (0.05) | 0.51 (0.05) | 0.41 (0.04) |
| **Kernel Cox** | 0.60  (0.07) | 0.19  (0.14) | 0.07 (0.07) | 0.62 (0.08) | 0.32 (0.13) | 0.79 (0.02) | 0.46 (0.13) | 0.61  (0.07) | 0.20  (0.15) | 0.08 (0.09) | 0.64 (0.08) | 0.31 (0.13) | 0.79 (0.02) | 0.47 (0.13) |
| **wSVM-KM** | 0.59  (0.04) | 0.14  (0.16) | 0.01 (0.06) | 0.61 (0.09) | 0.29 (0.13) | 0.89 (0.02) | 0.43 (0.13) | 0.58  (0.04) | 0.08  (0.14) | 0.01 (0.06) | 0.63 (0.08) | 0.29 (0.13) | 0.89 (0.02) | 0.43 (0.13) |
| **wSVM-Prop** | 0.57  (0.04) | 0.14  (0.15) | 0.01 (0.06) | 0.61 (0.09) | 0.27 (0.01) | 0.82 (0.02) | 0.42 (0.14) | 0.59  (0.04) | 0.08  (0.14) | 0.01 (0.06) | 0.63 (0.08) | 0.28 (0.01) | 0.82 (0.02) | 0.42 (0.14) |
| **pSVM-linear-KM** | 0.62  (0.08) | 0.21  (0.17) | 0.07 (0.08) | 0.65 (0.10) | 0.72 (0.04) | 0.81 (0.06) | 0.77 (0.05) | 0.61  (0.07) | 0.20  (0.14) | 0.08 (0.08) | 0.64 (0.09) | 0.72 (0.03) | 0.80 (0.06) | 0.78 (0.05) |
| **pSVM-linear-prop** | 0.61  (0.08) | 0.21  (0.17) | 0.07 (0.08) | 0.65 (0.10) | 0.71 (0.04) | 0.75 (0.06) | 0.71  (0.05) | 0.59  (0.07) | 0.18  (0.14) | 0.09 (0.08) | 0.64 (0.09) | 0.71 (0.04) | 0.74 (0.06) | 0.71  (0.05) |
| **pSVM-radial-KM** | 0.63  (0.04) | 0.11  (0.14) | 0.01 (0.05) | 0.62 (0.09) | 0.62 (0.02) | 0.83 (0.04) | 0.69 (0.17) | 0.59  (0.07) | 0.12  (0.16) | 0.11 (0.16) | 0.63 (0.08) | 0.62 (0.02) | 0.83 (0.04) | 0.69 (0.17) |
| **pSVM-radial-prop** | 0.60  (0.05) | 0.11  (0.15) | 0.01 (0.07) | 0.61 (0.09) | 0.59 (0.02) | 0.79 (0.05) | 0.61  (0.14) | 0.54  (0.09) | 0.08  (0.15) | 0.61 (0.54) | 0.62 (0.08) | 0.59 (0.03) | 0.79 (0.03) | 0.61  (0.14) |
| **LUPI-linear-KM** | 0.63  (0.08) | 0.22  (0.15) | 0.13 (0.09) | 0.62 (0.09) | 0.79 (0.04) | 0.69 (0.07) | 0.71 (0.04) | 0.63  (0.07) | 0.23  (0.15) | 0.11 (0.12) | 0.63 (0.07) | 0.79 (0.04) | 0.69 (0.07) | 0.71 (0.04) |
| **LUPI-linear-prop** | 0.60  (0.08) | 0.21  (0.15) | 0.12 (0.09) | 0.62 (0.09) | 0.75 (0.04) | 0.62 (0.07) | 0.65 (0.04) | 0.60  (0.07) | 0.22  (0.15) | 0.11 (0.12) | 0.63 (0.07) | 0.75 (0.04) | 0.63 (0.06) | 0.65 (0.04) |
| **inSVM-gradient** | 0.64  (0.07) | 0.25  (0.15) | 0.06 (0.07) | 0.67 (0.09) | 0.83 (0.03) | 0.81 (0.06) | 0.81 (0.04) | 0.63  (0.07) | 0.25  (0.13) | 0.11 (0.09) | 0.67 (0.08) | 0.83 (0.03) | 0.81 (0.06) | 0.81 (0.04) |
| **inSVM-averaging** | 0.64  (0.07) | 0.24  (0.15) | 0.06 (0.07) | 0.67 (0.09) | 0.82 (0.03) | 0.83 (0.06) | 0.82  (0.04) | 0.64  (0.07) | 0.26  (0.14) | 0.11 (0.08) | 0.67 (0.08) | 0.82 (0.03) | 0.83 (0.06) | 0.83  (0.04) |
